# On the Race for More Stretchable and Tough Hydrogels

**DOI:** 10.3390/gels5020024

**Published:** 2019-04-28

**Authors:** Santiago Grijalvo, Ramon Eritja, David Díaz Díaz

**Affiliations:** 1 Institute for Advanced Chemistry of Catalonia (IQAC, CSIC), Jordi Girona 18-26, E-08034 Barcelona, Spain; sgrgma@cid.csic.es (S.G.); recgma@cid.csic.es (R.E.); 2 Networking Centre in Bioengineering, Biomaterials and Nanomedicine (CIBER-BBN), Jordi Girona 18-26, E-08034 Barcelona, Spain; 3 Instituto de Productos Naturales y Agrobiología del CSIC, Avda. Astrofísico Francisco Sánchez 3, 38206 La Laguna, Tenerife, Spain; 4 Institut für Organische Chemie, Universität Regensburg, Universitätsstr. 31, 93053 Regensburg, Germany

**Keywords:** composite hydrogels, double cross-linking hydrogels, hydrogen bonds, hydrophobic association, self-healing, stretchability, synthetic polymers, toughness

## Abstract

Hydrogels are tridimensional networks that are able to retain important amounts of water. These soft materials can be obtained through self-assembling processes involving either hydrophilic molecules or polymers, allowing the formation of the corresponding covalently and physically cross-linked networks. Although the applicability of hydrogels in biomedicine has been exponentially growing due to their biocompatibility and different responses to stimuli, these materials have exhibited the particular feature of poor mechanical strength, and consequently, are brittle materials with low deformation. Due to this reason, a race has started to obtain more stretchable and tough hydrogels through different approaches. Within this context, this review article describes the most representative strategies and examples involving synthetic polymers with potential for biomedical applications.

## 1. Introduction

The progress and growing interest experienced in the field of hydrogels in the last decade has helped to speed up multiple applications ranging from biomedicine to industrial processes [1,2,3]. Hydrogels are tridimensional (3D) networks made of hydrophilic polymers, which can swell in aqueous solvents and maintain their 3D structure properties when retain a large amount of water by both chain entanglement and cross-linking methods (e.g., physical or chemical) between individual polymer chains of the material. These properties allowed hydrogels to become promising materials capable of mimicking certain physical, chemical, and electrical features of many biological tissues [4]. There are different ways to classify hydrogels which can depend on several aspects, such as their physical structure, preparation methods, ionic charges, and polymer origin [1,5]. The nature of the hydrogel cross-linking can play an important role in the way in which they response to certain stimuli by exhibiting transition changes from the solid to liquid state (gel-to-sol). This has allowed physical and chemical stimuli (e.g., light, pH, temperature, magnetic fields, etc.) to be used in many different biomedical applications including controlled drug delivery, diagnostics, cell encapsulation or tissue engineering [6].

Synthetic hydrogels can be obtained from different chemical methods, in particular, using thermal or photo-initiated polymerizations [7]. These strategies led to some success in the development of numerous advanced materials with improvements in mechanical strength, biodegradability, drug release rates, and cross-linking density. These hydrogels have been preferentially made up of monomers like 2-hydroxyethyl methacrylate, acrylamide (AAm), vinyl acetate, and ethylene glycol, among others [8]. Extensive knowledge about their synthetic methods and reproducibility, as well as material behaviors, contributed to synthesizing tailored hydrogels that were in multiple fields and integrated into many biomedical applications.

In addition to synthetic hydrogels, natural polymers are obtained from polysaccharides and fibrous proteins and may represent the most valuable alternative to synthetic materials because of their biocompatibility and biodegradability properties [9]. Natural, and in some regards, conventional synthetic hydrogels, face some limitations with respect to their low tensile strength when in contact to aqueous solutions [10] despite being successfully used in multiple applications by loading small molecule drugs [11], colloidal systems [12], and gold nanoparticles [13]. Therefore, the swelling process does not appear to be as effective as it could be, which results in reduced mechanical strength and limited mechanical properties leading to the fracture of the entire body when subjected to local stress. This important limitation can considerably reduce the applicability of hydrogels as potential biomaterials [14], though many strategies have been implemented in order to improve their mechanical behaviors like stretchability, toughness, and self-healing properties [15,16,17,18,19,20].

This limitation has drawn attention to designing materials with enhanced stretchable and toughness properties [21,22]. A hydrogel is considered to be tough when fracture energy and tensile stress oscillates between 10^2^–10^3^ J m^−2^ and 0.1–1 MPa, respectively. According to dissipation energy models, different theoretical models were proposed with the aim to rationalize both elasticity and resistance mechanisms when applied a shear-force into artificial soft materials but maintaining their high elasticity. Most of these examples include double network hydrogels, topology hydrogels, tetra-polyethylene glycol (PEG) hydrogels, macromolecular microspheres, and the use of noncovalent or covalent composite hydrogels. In this review, we aim to describe the most recent and relevant strategies described for achieving stretchable and tough hydrogels from synthetic sources putting special emphasis on designing double-network materials as well as the influence made by clay minerals, inorganic nanoparticles and metal ions on the mechanical properties when these cross-linkers are part of polymer networks. Additionally, recent strategies developed for the preparation of supramolecular hydrogels and the potential applicability of such composite materials in biomedicine are also discussed. 

## 2. Stretchable and Tough Hydrogels from Synthetic Polymers

One of the main limitations that regular hydrogels must face is their brittleness when they are swollen in water. This reduction in their mechanical properties allowed the design of new strategies for increasing the mechanical strengths of hydrogels, including toughness and stretchability. In this regard, it is worth mentioning that interpenetrating polymer network (IPN) [23] and double-network (DN) [24,25] hydrogels are important types of materials that have exhibited meaningful properties like mechanical strength and toughness. IPN hydrogels are made up of two or more polymer networks, which are cross-linked by electrostatic interactions rather than covalent bonds. This strategy allowed these materials to be used in a plethora of biomedical applications, especially as drug delivery systems [23]. Synthetic efforts in increasing the mechanical properties of IPN hydrogels have been reported and had a significant role in tissue engineering and other medical applications [26,27,28].

DN hydrogels are a type of IPN hydrogels which are made up of mainly water (up to 90 wt%) and a combination of two polymer networks that differ in their composition. Thus, this experimental procedure involving cross-linked polyelectrolytes and neutral polymers allowed the preparation of interesting materials with certain elastic modulus of 0.1–1.0 MPa and fracture energies of 100–1000 Jm^−2^. In addition, such DN hydrogels have also displayed high biocompatibility degrees, giving rise to artificial tissues capable of mimicking natural cartilage [29] and other important biomedical applications [20,30,31]. Some of the strategies used to enhance the physical properties of hydrogels are listed below. 

### 2.1. Nanocomposite Hydrogels

**Figure 1:**
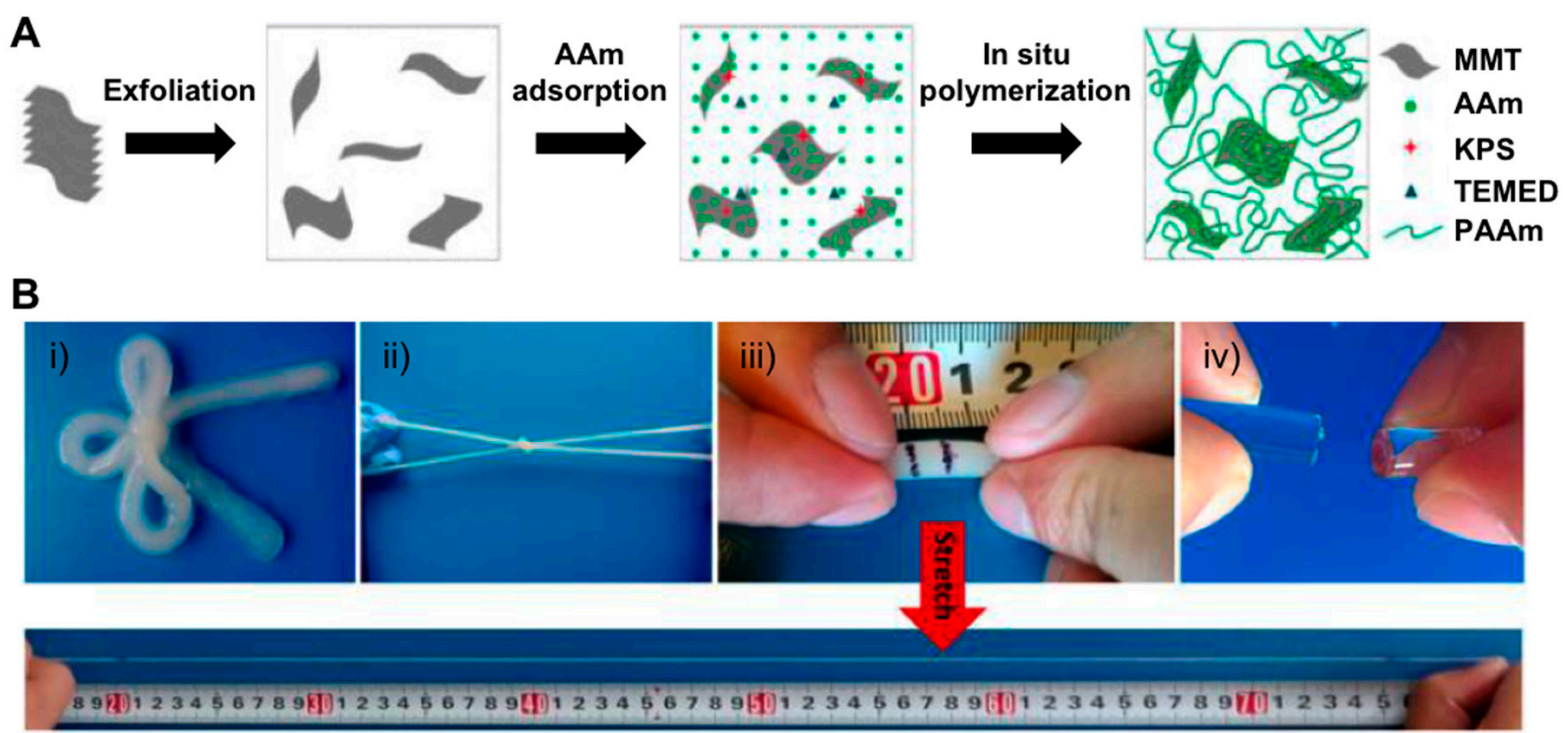
(**A**) Schematic illustration of the synthesis of hydrogels containing montmorillonite (MMT) and poly(acrylamide) (PAAm). (**B**) Photographs of hydrogels: (**i**–**iii**) Hydrogel (10 wt.% of clay with respect to AAm) bow-tie and that highly stretched to a strain of 11,000%. (**iv**) Covalently cross-linked PAAm hydrogel with 0.1 mol% *N*,*N’*-methylene-bis(acrylamide) (MBAAm) shows a brittle fracture at low strain. Adapted with permission from Reference [39]. Copyright 2015 American Chemical Society.

Nanocomposite hydrogels (NCHs) are hybrid materials in which nanoparticles can be part of the hydrogel network [32]. A good number of nanoparticles have been used and combined with polymer networks (e.g., graphene [33], carbon nanotubes [32,33], polymeric nanoparticles [32], ceramic-inorganic [34], clay minerals [35], and metal-oxide/metal nanoparticles [36]), yielding materials with advanced properties and functionalities that have been able to be employed in several biomedical and biotechnological applications. NCHs are easily obtained with high yield by in situ free-radical polymerization approaches under mild conditions. This method enables the preparation of smart hydrogels with high toughness and superior optical properties. Since the Haraguchi’s pioneering work who reported the NCHs synthesis from poly(*N*-isopropyl acrylamide (PNIPAm) using synthetic clay as a multifunctional cross-linker for the first time [37], these materials exhibited an exponential growth in tissue engineering and drug delivery applications [32,38]. A key element in the preparation of NCHs is the possibility of preparing self-healing hydrogels with enhanced toughness and strength properties. Gao et al. developed a preparation that involved exfoliated sodium montmorillonite (NaMMT) nanosheets in combination with AAm monomers for the first time [39]. The synthetic strategy followed by the authors was based on carrying out the adsorption of the corresponding monomer and subsequent in situ polymerization (potassium peroxodisulfate (KPS) and *N*,*N*,*N*,*N*-tetramethylethylenediamine (TEMED) are used as initiator and catalyst), yielding the anticipated NCHs with clay contents of up to 50% (Figure 1A). The authors confirmed the hydrogel formation was stabilized through hydrogen bonding after measuring hydrogel stabilities in the presence of water and urea. Additionally, FT-IR corroborated the presence of H-bonding between hydroxyl and amide groups. The presence of MMT as a noncovalent cross-linker within the polymer network (2.2–5.7 mol m^−3^, approximately) increased both a fracture elongation and a fracture toughness of the material up to 11,800% and 10.1 MJ m^−3^, respectively (Figure 1B). The authors also observed that materials were able to dissipate energy in mild conditions, as well as the ability to fully recover their original morphology, when subjected to deformation under cyclic tensile loading/unloading experiments. Interestingly, the presence of clay nanosheets within the 3D network facilitated the expected noncovalent interactions with polymer chains, which in turn afforded the self-healable nature of the material after drying-re-swelling procedures.

**Figure 2:**
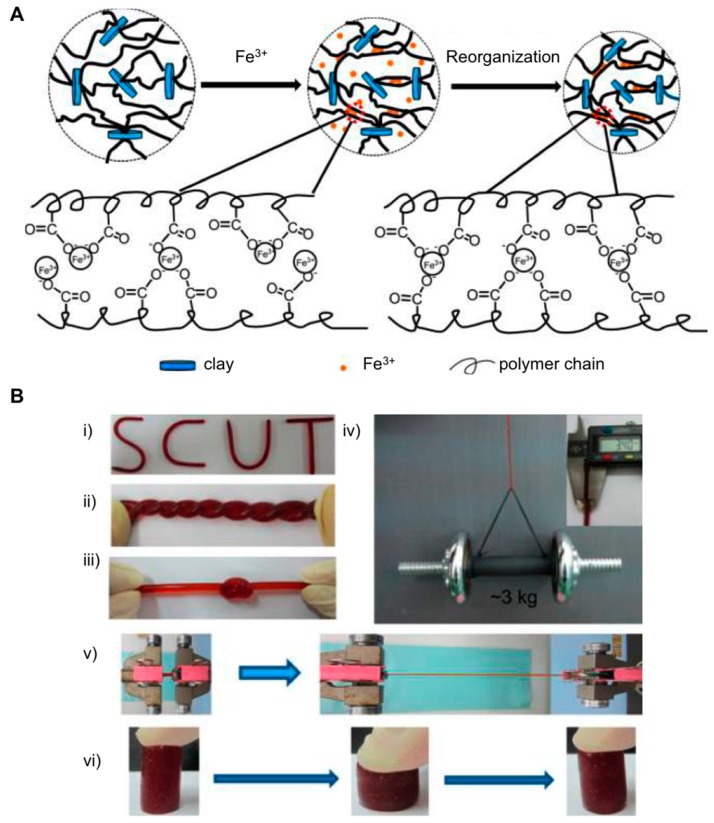
(**A**) Preparation of dual physically cross-linked (DPC) hydrogels: In situ polymerization of AAm and acrylic acid (AAc) in the presence of clay to form physically cross-linked network; the interaction of –COO– groups of polymer chains with Fe^3+^ and then reorganization of Fe^3+^ coordinates to introduce Fe^3+^ coordination physically cross-linked points. (**B**) DPC hydrogel can be (**i**) bended, (**ii**) twisted into double-helix structure, and (**iii**) knotted. (**iv**) DPC hydrogel with the diameter of 3.4 mm lifts up dumbbell of ca. 3 kg in weight. (**v**) High strain is applied to the DPC hydrogel. (**vi**) DPC hydrogel can be strongly compressed and quickly recovered its shape upon the removal of compression force. Adapted with permission from Reference [40]. Copyright 2016 American Chemical Society.

In addition to exfoliated MMT, Laponite XLG was also used by Hu et al. as inorganic clay nanosheets to engineer dual physically cross-linked (DPC) hydrogels [40] in combination with iron (Fe^3+^) ions [41]. These materials showed two differentiated cross-linking points: The first one involved the coordination of Fe^3+^ ions and carboxylate groups of poly(acrylamide-*co*-acrylic acid (P(AAm-*co*-AAc)), giving rise to the primary cross-linking junctions, whereas clay nanosheets promoted the generation of hydrogen bonds with P(AAm-*co*-AAc) polymer chains, producing the second cross-linking section (Figure 2A). This strategy based on the presence of noncovalent bonds remarkably improved the mechanical properties of DPC hydrogels than those obtained from covalently connected networks as they tend to have poor recoverability and low resistance after loading cycles. In this particular case, the resultant physically DPC hydrogels displayed excellent properties like high tensile strength (ca. 3.5 MPa), exceptional toughness values (ca. 49 MJ m^−3^), large elongation (ca. 21 times), and self-recoverability (ca. 65% recovery) from external damages at the optimal formulation tested (Figure 2B).

Recently, Quin et al. engineered hydrogels by micellar copolymerization of two hydrophilic monomers, like AAm and acrylic acid (AAc), in combination with a hydrophobic monomer, like stearyl methacrylate (SMA), following the same DPC approach [42]. This approach enabled the synthesis of poly(stearyl methacrylate) and facilitated the formation of the first cross-linking point through strong hydrophobic interactions. Finally, the dual-cross-linked hydrogel was developed taking advantage of the ionic coordination bonds between Fe^3+^ ions and the corresponding carboxylic groups obtained previously. The authors showed that mechanical properties of the hydrogel could be widely modulated by regulating the total monomer concentration (w/v), hydrophobic association density (e.g., mass ratio variation of SMA, AAm and AAc), and the resultant ionic coordination bonds (e.g., FeCl_3_ concentration) generated in the material. The optimal formulation yielded physical hydrogels, which showed enhanced mechanical properties, such as good self-recovery (ca. 83%), after applying cyclic tensile experiments at 400% strain for 4 h, high tensile strength (ca. 6.8 MPa), excellent elongation ability (ca. 1000%), and toughness (53 MJ m^−3^).

Other interesting composite hydrogels involving physically cross-linked networks were recently engineered. The strategy developed by Zhang et al. was directed through electrostatic interactions between quaternized cellulose nanocrystals (Q-CNCs) and the negatively charged polymer side chains of poly(acrylic acid-*co*-acrylamide) (P(AAc-*co*-AAm) [43]. The resulting cross-linked network was reinforced by adding a ferric chloride solution, which was able to interact with the COO– groups and thus afforded the compaction of the polymer network by ionic coordination of Fe^3+^ ions. This strategy enabled the synthesis of dual-cross-linked (D-Gel) hydrogels as well as mono-cross-linked (M-Gel) hydrogels, which were also prepared for comparison purposes. As expected, the presence of Fe^3+^ ions displayed remarkably high toughness and stiffness and maintained the integrity of D-gels in the presence of several stimuli. Notably, mechanical properties, such as tensile strength and elongation at break of the prepared hydrogels (M-Gel and D-Gel), depended on both the Q-CNCs content and polymer chain concentration. This allowed the authors to prepare dual cross-linked hydrogels 340 times tougher than in the case of mono-cross-linked hydrogels. In both cases, such composite materials exceeded the mechanical properties exhibited by plain hydrogels. Finally, the same authors also studied the self-recovery ability of the cross-linked hydrogels. The results showed that they were able to recover their original morphology within 2 h and this property was dependent on the recovery conditions with the FeCl_3_ aqueous solution being much faster than in air and water, respectively.

**Figure 3:**
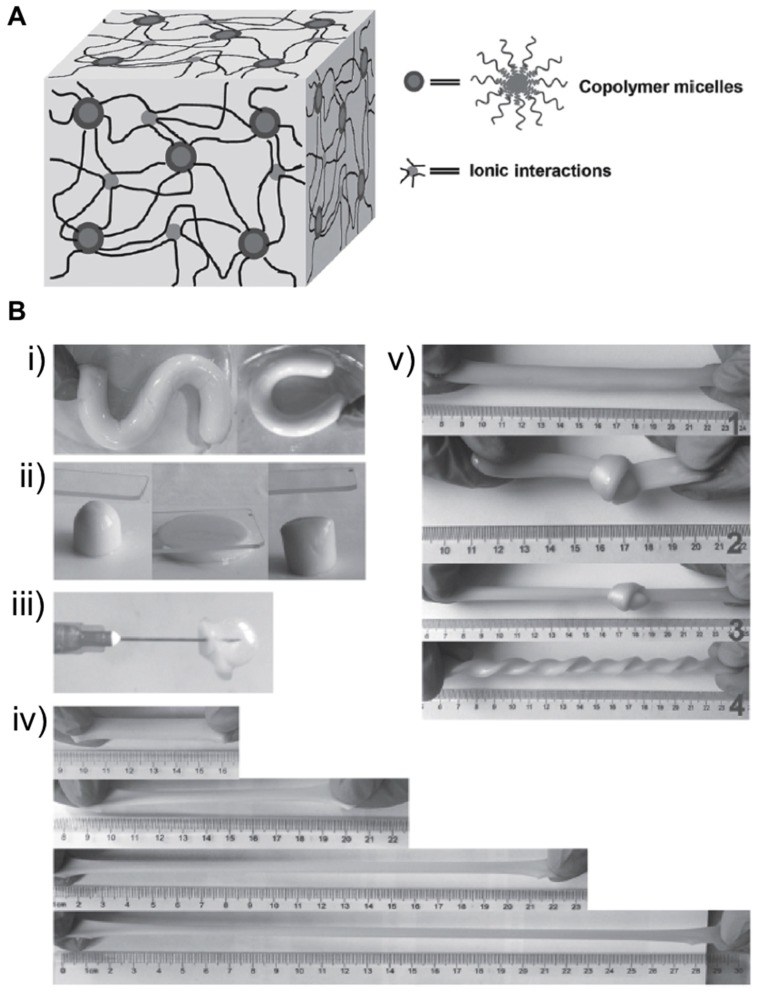
(**A**) Illustration of the double cross-linked (DC) hydrogel network. (**B**) Photographic images of the DC hydrogels: (**i**) An as-prepared hydrogel is bent into “S” shape and “C” shape. (**ii**) A DC hydrogel is pressed using a glass slide. From left to right, the images show the original, pressed, and released states, respectively. (**iii**) A balloon is made by blowing the DC hydrogel with a syringe. (**iv**) A DC hydrogel strip (water content 65%) is stretched to ~200%, ~370%, and ~540% of its original length. (**v**) From top to bottom, the images show a DC hydrogel strip, the hydrogel strip is knotted, the knotted hydrogel strip is stretched, and the hydrogel is unknotted and twisted. Adapted with permission from Reference [44]. Copyright 2017 Wiley-VCH.

Double cross-linked (DC) hydrogels were also prepared by combining triblock co-polymers micelles and Fe^3+^ (Figure 3A). This strategy allowed Zhou et al. to prepare flexible, tough, and self-healable materials, which resulted in an alternative to the preparation of artificial soft materials with important mechanical properties like flexibility, toughness, and self-healing [44]. The authors first obtained micelles from chemically modified Pluronic F127 (PF127), which acted as a chemical cross-linking agent and carried out a one-pot radical polymerization reaction involving AAc, PF127, Fe^3+^ ions, and ammonium persulfate. This process gave rise to brown and opaque dual cross-linked hydrogels that could be twisted and bent without fracturing (Figure 3B). Notably, these materials were able to withstand deformation processes of 600% with ultimate tensile stress of 210 kPa when the molar ratio of the system Fe^3+^/AA was fixed at 0.5 mol%. When this relationship was increased to 1.25 mol%, this ultimate stress proportionally increased, though the ultimate strain displayed a remarkable decrease. Additionally, the material with 65 wt.% of water content was stretched up to 540% its original length. As described in other examples, the presence of ionic interactions between Fe^3+^ ions and polymer chains within the polymer network displayed the capacity to efficiently dissipate energy throughout their polymeric network and be a self-healing material (73% of healing efficiency). However, the mechanical strength recovery of this material was limited up to 40%, according to the authors. This property involving the synthesis of micellar polymers may be a good strategy for the development of artificial soft tissues like skin, tendons, and muscles, among others.

**Figure 4:**
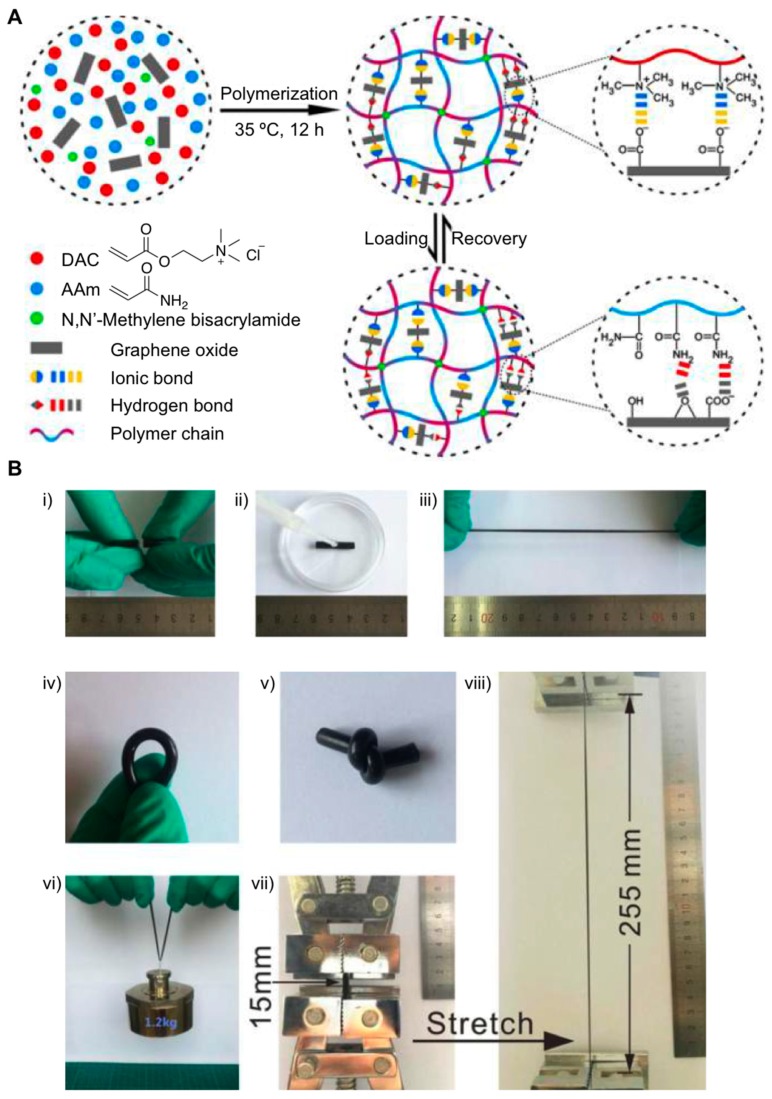
(**A**) Schematics of preparation of hydrogel copolymerization of AAm, DAC and MBAAm in the presence of graphene oxide (GO) along with a proposed molecular mechanism of the loading and recovery process. The ionic bonds and hydrogen bonds contribute to the high mechanical properties and self-healing properties. (**B**) (**i**–**iii**) Self-healing properties of the hydrogels: (**i**) A pristine cylinder of the A_1_D_2_G_1_ sample was cut in half; (**ii**) the two-halves were simply made to contact, and a drop of water as dropped on the cut surface; (**iii**) after standing for hours, the sample can be stretched to a large strain by hand. (**iv**–**viii**) A_1_D_2_G_1_ hydrogels can be (**iv**) bent, (**v**) knotted, (**vi**) loaded, and (**vii**) stretched. Nomenclature of hydrogels: A*_x_*D*_y_*G*_z_*, where A, D, and G represent AAm, DAC, and GO, respectively; *x* and *y* refer to the mass ratio of AAm and DAC; *z* represents the GO content. Adapted with permission from Reference [48]. Copyright 2017 American Chemical Society.

The use of graphene has become an interesting strategy to obtain materials with improved mechanical properties in terms of stretch, chemical stability, electrical and thermal conductivity, as well as good self-healing abilities [45,46,47]. Interesting results were obtained by Pan et al. [48] after carrying out free-radical polymerization reactions involving AAm, 2-(dimethylamino)ethylacrylatemethyl chloride (DAC), and *N*,*N*′-methylene bisacrylamide (MBAAm) in the presence of graphene nanosheet contents at pH value of 10 (Figure 4A). The resultant solutions showed high zeta potential values confirming the colloidal stability of the dispersion. After conducting the polymerization reaction at 35 °C, the authors finally obtained poly(AAm-*co*-DAC)/graphene hydrogels with water contents close to 30 wt.%. The composite characterization by FITR and Raman spectroscopic techniques also confirmed the presence of electrostatic interactions between the oxygen groups of graphene nanosheets and the amine groups of polyacrylamide polymer chains. Several hydrogels containing mass ratios of AAm and DAC (1:0.5, 1:1 and 1:2), as well as graphene (0, 0.5, 1.5 and 2 wt.%), were prepared. The authors confirmed the fracture stress and strain of the composites increased up to 564.1 KPa and 1608%, respectively, as the graphene concentration was raised 1.0 wt.%. Further studies based on modulating the amount of AAm and DAC allowed the authors to achieve optimal formulations that remarkably improved the mechanical properties of these hydrogels. In this regard, excellent results were obtained in the case of hydrogels containing AAm (1.0 wt.%), DAC (0.5 wt.%) and graphene (2.0 wt.%). This key combination yielded a high Young’s modulus (ca. 1056 KPa) and high tensile strength (ca. 2.1 MPa) at elongation of ca. 810%, as well as high toughness (ca. 9.3 MJ m^−3^) (Figure 4B). In addition to these outstanding properties, the presence of graphene in the hydrogel composite also showed additional features like being able to go back to its original size quickly (less than 10 s) and fatigue resistance, as well as self-healing abilities both in the presence and absence of water. It was observed that the hydrogel morphology was almost stable without causing any damage to the composite structure after going through 30 compressive loading-unloading cycles.

**Figure 5:**
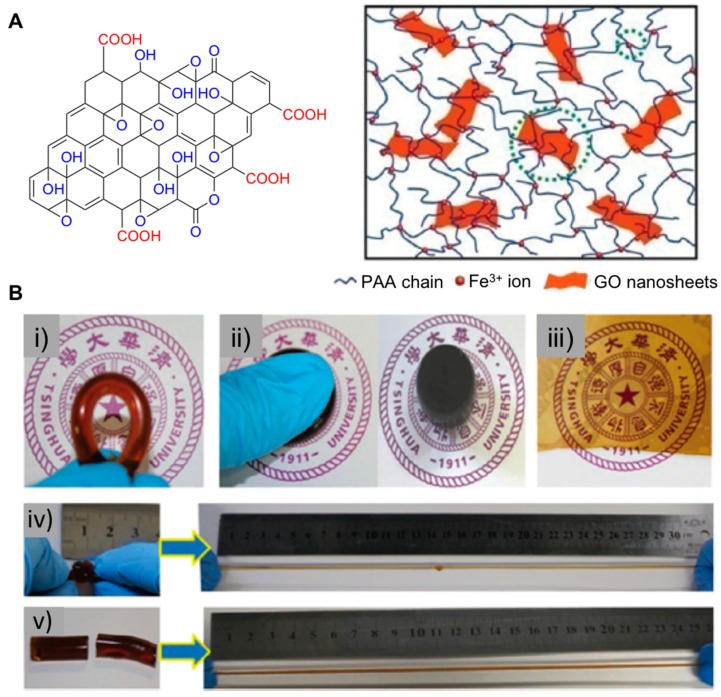
(**A**) ***Left***: Chemical structure of GO. ***Right***: A scheme of the 3D network structure of GO-PAA nanocomposite hydrogels facilitated by Fe^3+^ ions with dual cross-linking effects (the smaller green dotted circle represents the first cross-linking points that are Fe^3+^ ions creating ionic cross-linking among PAA chains, and the larger green dotted circle represents the second cross-linking points that are GO nanosheets linking PAA chains through coordination). (**B**) Photographs of GO-PAA nanocomposite hydrogels under different conditions demonstrating their excellent structural diversity and processability. The GO-PAA nanocomposite can be (**i**) bent, (**ii**) compressed, (**iii**) modulated into a semitransparent film, (**iv**) stretched over 30 times from a knotted state, and (**v**) stretched over 20 times after being healed (45 °C, 48 h) from a cut-off state. Adapted with permission from Reference [49]. Copyright 2015 Royal Society of Chemistry.

In addition to combining polymers and graphene oxide directly, introducing metal ions (e.g., Ca^2+^ and Fe^3+^) resulted in the preparation of DC networks, which yielded interesting stretchable and self-healable materials with functional properties. Following similar synthetic strategies based on double network mechanisms, Zhong et al. prepared poly(acrylic acid) (PAAc)–graphene oxide (GO) nanocomposite hydrogels by adding Fe^3+^ as a cross-linker through in situ one-pot radical polymerization [49] (Figure 5A). The optimal contents of Fe^3+^ (0.5 mol%) and GO (0.5 wt.%) were selected. These materials exhibited good recovery properties maintaining their original morphology after being stretched due to the presence of the DC networks based on ionic interactions among PAA polymer chains and graphene nanosheets. In this regard, materials were able to dissipate energy transferring the stress content throughout the polymer matrix. Additionally, superior and robust mechanical properties were achieved in terms of elongation at break (2980%) and high toughness (777 KPa), as well as self-healing properties (tensile strength of 495 kPa) and work of extension at fracture (i.e., work required for fracturing a material per unit volume) of 11.9 MJ m^−3^ (Figure 5B). These promising results highlighted the powerful influence of DC networks by synthesizing composite materials with potential use as injectable implants, artificial skin, and other biomedical engineering applications.

**Figure 6:**
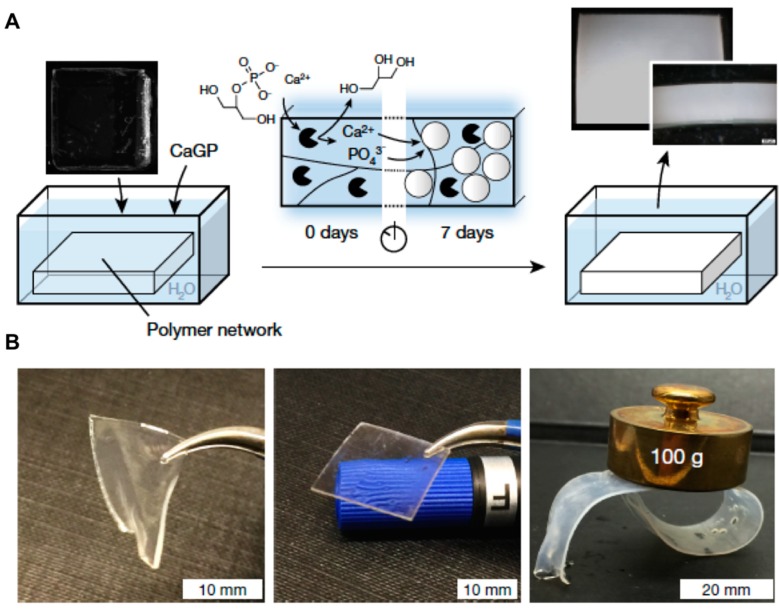
(**A**) Scheme of enzyme-induced bulk calcification of different polymer networks. Black pie shapes represent the enzyme, and the white circles represent the formed amorphous structures. The photographs show PDMA-*l*-TEG networks before (***left***) and after seven days (***right***) of mineralization. (**B**) Images of swollen PDMA-*l*-TEG network containing 10 wt.% EDPOA after seven days in water (***left***) or in calcification solution (***middle***). A circular-shaped swollen PDMA-*l*-TEG network containing 2 wt.% EDPOA and calcified for seven days, here loaded with a 100-g weight (***right***). Adapted with permission from Reference [50]. Copyright 2017 Nature Research.

Other strategies for modulating the mechanical properties of synthetic hydrogel networks were recently reported by Rauner et al. [50]. This approach includes the entrapment of alkaline phosphatase (via photopolymerization) within three biocompatible synthetic polymers (e.g., triethylene glycol dimethacrylate (PHEA-*l*-TEG); poly-*N*,*N*-dimethyl acrylamide-*l*-TEG (PDMA-*l*-TEG), and polyacrylamide-*l*-*N*,*N*′-methylenebis-(acrylamide) (PAAm-*l*-MBAm)). Then, 2-glycerol phosphate (CaGP) calcification solution is added enabling the enzyme to catalyze the dephosphorylation of the CaGP. This process allows calcium and phosphate to remain in the polymer network in the form of amorphous structures. The film turned from transparent to cloudy, suggesting that mineralization process took place (Figure 6A). A full characterization of the three hydrogels by electron microscopy confirmed the presence of the inorganic content in their 3D network (8, 72, and 70 wt.% for PHEA-*l*-TEG, PDMA-*l*-TEG, and PAAm-*l*-MBAm, respectively). The authors noticed that as the mineralization degree increased, hydrogel stiffness varied proportionally, reaching Young’s values (elastic) of 73 and 155 MPa as well as achieving fracture energies of 65 and 763 J m^−2^ for PDMA-*l*-TEG and PAAm-*l*-MBAm hydrogels, respectively. Notably, the authors also explored the creation of additional electrostatic interactions by introducing different amounts of phosphonate groups like ethyl-2-[4-(dihydroxyphosphoryl)-2-oxabutyl]-acrylate (EDPOA) into the prepared hydrogels. This strategy activated the interaction with calcium phosphate nanostructures, and consequently, it was also able to tune and modulate the mechanical properties of the nanocomposites, and in particular, the PDMA-*l*-TEG hydrogel. The presence of phosphonate groups provided comparable Young’s modulus, although this hydrogel increased its toughness properties (485 J m^−2^) with a 10 wt.% of EDPOA content (Figure 6B). Interestingly, when the EDPOA concentration was increased, it produced homogeneous structures and reduced the aggregate size producing more transparent composite hydrogels (ca. 92% transmittance at 700 nm). In addition to using calcium phosphate, the authors also showed that glucose-6-phosphate (G-6-P) could mediate the mineralization process with similar efficiencies. This property conferred the possibility of using these materials in biomedical processes like regenerative medicine and drug delivery because of the resulting composites may be compatible with biological systems [51].

**Figure 7:**
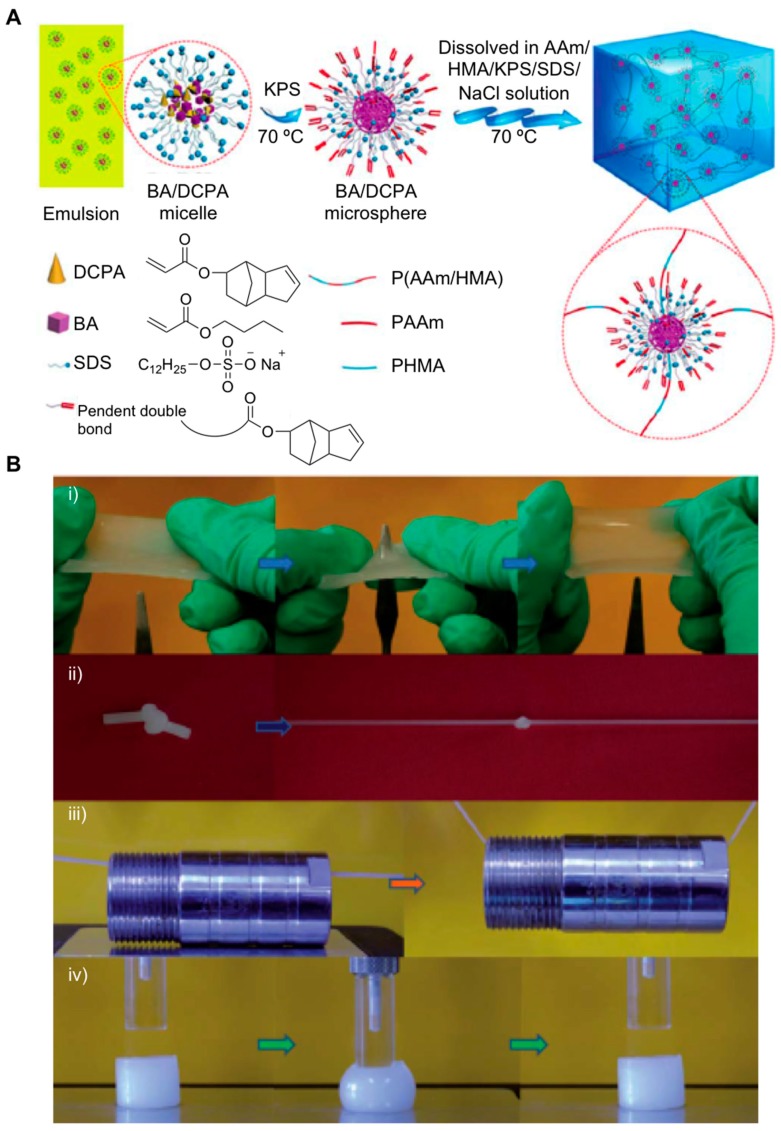
(**A**) Structure and formation mechanism of cross-linked P(AAm/HMA) hydrogels. (**B**) Demonstration of the toughness of macromolecular (MM) cross-linked P(AAm/HMA) hydrogels through (**i**) stabbing; (**ii**) knotted stretching; (**iii**) loading and (**iv**) compressing experiments (MM content was 1.0 wt.% of added monomers and the diameter was 349 nm after adding monomers). Adapted with permission from Reference [55]. Copyright 2016 Royal Society of Chemistry.

The use of macromolecular microspheres (MMSPH) like cross-linkers represents another type of strategy for modulating the mechanical behavior of hydrogels [52,53,54]. This approach, which was developed by Ren et al., first involves an emulsion polymerization reaction in the presence of sodium dodecyl sulfate (SDS) between butyl acrylate (BA) and dicyclopentyl acrylate (DCPA) in order to avoid the MMSPH aggregation [55] (Figure 7A). Additionally, particles size could be controlled by adding different amounts of both emulsifier and electrolytes. Second, a radical polymerization reaction triggered in water after combining AAm and hexadecyl methacrylate (HMA) which gave rise to the corresponding composite P(AAm/HMA)-MMSPH hydrogels. The mechanical behavior of these materials showed excellent results in terms of fracture stress (0.555 MPa), which were based on several parameters like particle size (ca. 349 nm of diameter) and fracture strain (5533%), as well as good perforate resistance attributes when compared to unmodified hydrogels (Figure 7B). This response to external stimuli was attributed not only to the presence of the MMSPH within the hydrogel network, but also to the formation of hydrophobic entanglements among HMA polymer chains leading to the corresponding micellar structures. The authors hypothesized that this kind of physically cross-linking bonds may help to dissipate the energy caused by hydrogel deformation during strain-stress cycles demonstrating a novel strategy for obtaining toughened materials with potential applications for biomaterials.

The concentration of nanoparticles and clays inside polymeric networks is an important parameter to take into account [56,57]. Depending on their amount, the mechanical properties of composites can vary in terms of strength and toughness, among others. In addition to concentration, the diameter size of the particles can also play a pivotal role in the mechanical properties of the composite hydrogels. In this regard, while size of the most particles within composite hydrogels may be bigger than 60 and 800 nm for nanoparticles and clays, Sun et al. [16] designed a methodology involving the use of nonaggregated calcium hydroxide [Ca(OH)_2_] spherulites with diameters less than 5 nm in solution. These nanoparticles were used as cross-linkers for polyacrylamide hydrogels after promoting an acid-base reaction between Ca(OH)_2_ and ammonium peroxydisulfate. This gelation process was studied in-depth and it was confirmed that vacuum conditions and low temperatures (0.01 atm, 0 °C) were indispensable preconditions when starting the polymerization reaction. This strategy enabled the synthesis of materials with a homogeneous distribution and high density of spherulites within the 3D network. The mechanical properties showed outstanding values regarding stretch at rupture (121 KPa) and stress ratio (430 KPa) when the concentration of spherulites did not exceed 40 ppm. This remarkably improved the properties of conventional hydrogels containing 3 wt.% of clay. Interestingly, when the concentration of such inorganic components was increased up to 200 ppm, the composite hydrogel was able to return its original size (ca. 90%) after carrying out a compressive stress of 100 MPa and being stretched over four cycles.

**Figure 8:**
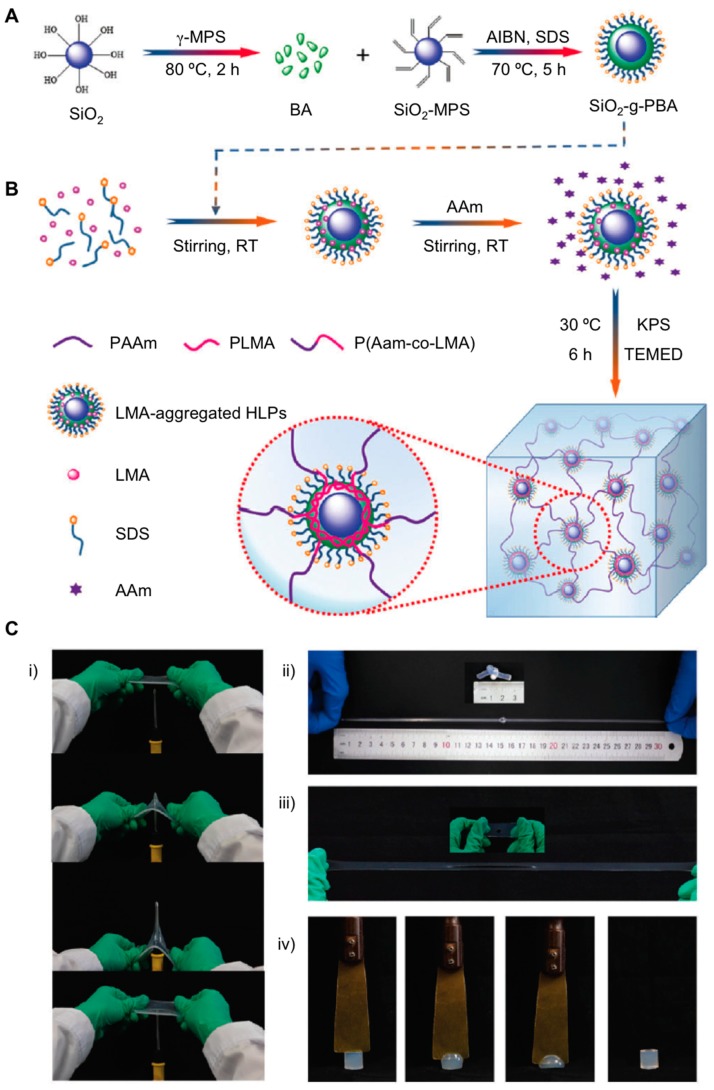
(**A**) Formation mechanism of P(AAm-*co*-LMA)-HLPs hydrogels reinforced by core-shell SiO_2_-*g*-PBA hybrid latex particles. MPS = 3-methacryloxypropyltrimethoxysilane; AIBN = 2,2′-azobis(2-methylpropionitrile). (**B**) P(AAm-*co*-LMA) hydrogels were prepared by using SiO_2_-*g*-PBA core–shell HLPs as dynamic crosslinking centers for efficient aggregation of hydrophobic segments. (**C**) Display of the mechanical properties of P(AAm-*co*-LMA)-HLPs hydrogels: (**i**) stabbing; (**ii**) and (**iii**) large stretching with a knot and a hole; and (**iv**) compression with a knife. Adapted with permission from Reference [59]. Copyright 2017 Royal Society of Chemistry.

The use of silica nanoparticles (SiO_2_) as cross-linking particles offers another alternative way for preparing composite hydrogels [58]. This is mainly due to the fact that SiO_2_ has displayed innate high modulus and surface area, which is prompted to be functionalized. Poor self-recovery properties and exiguous fatigue resistance are some of the features found when hydrogels are usually made of chemically linked SiO_2_ particles. To overcome these drawbacks, Xia et al. successfully engineered a mixture of inorganic-organic materials based on SiO_2_-*g*-poly(butyl acrylate) hybrids latex particles (SiO_2_-*g*-PBA-HLP) by carrying out a mini-emulsion polymerization approach [59] (Figure 8A). The diameter and morphology of the particles were analyzed and characterized by dynamic light scattering (DLS) and transmission electron microscopy (TEM) (124.2 nm). The use of lauryl methacrylate (LMA) initiated an aggregation process in the presence of such silica hybrid particles and this was stabilized with the aid of surfactants. This strategy allowed the authors to synthesize poly(acrylamide-*co*-lauryl methacrylate) (P(AAm-*co*-LMA)) cross-linked with SiO_2_-*g*-PBA-HLP particles by activating aggregation processes and triggering hydrophobic interactions between polymer side chains of the composite hydrogel (Figure 8B). Extensive mechanical tests were studied and demonstrated good tensile strength with fracture stress and fracture strain (stretch) of 1.48 MPa and 2511%, respectively, as well as superior toughness (12.62 MJ m^−3^) when SiO_2_ particles were part of the synthetic hydrogel network. These superior values improved 2.1 (0.61 MPa) in tensile strength and 1.5 (1785%) in fracture strain, respectively, when poly(AAm-*co*-LMA) was used. Additionally, this strategy also demonstrated the capacity of providing materials with anti-fatigue, puncture resistance and self-recovery properties in accordance with a good number of loading-unloading cycle measurements (Figure 8C). In this way, the strategy followed by the authors may offer a promising approach for the use of such soft materials like artificial tendons, cartilages, and other biomedical applications. 

### 2.2. Supramolecular Hydrogels

Supramolecular hydrogels have been the subject of study for the last decade in numerous applications [60]. Unlike chemically cross-linked hydrogels in which their polymeric chains are permanently interconnected through covalent bonds, the presence of ionic interactions within the polymer network leads to the formation of supramolecular materials. These kinds of interactions can occur either between polymer chains or low molecular weight gelators (LMWG) through π–π stacking, host-guest complexation, metal-ligand coordination, hydrogen bonding, or protein interactions in aqueous media. The modulation and use of these parameters helped tune the cross-linking density, therefore modifying the mechanical properties of these kinds of hydrogels. The presence of H-bonding electrostatic interactions has made improvements in the mechanical behavior of a good number of materials through the cooperative action of the entire polymeric network in aqueous solutions [61]. 

**Figure 9:**
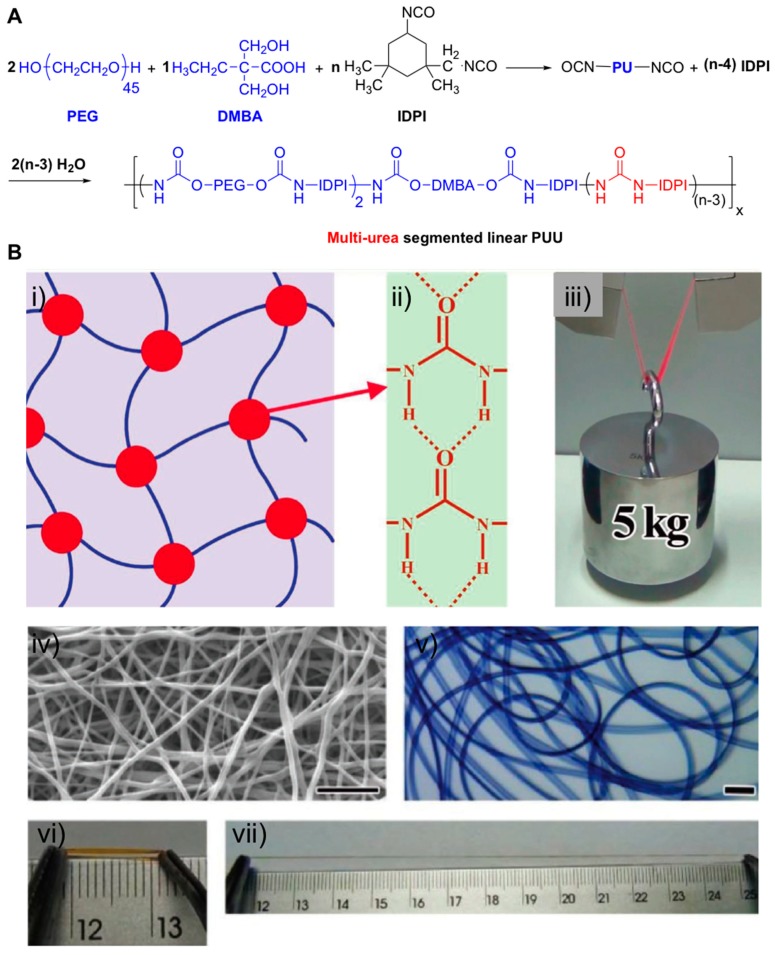
(**A**) Synthetic procedure of the multi-urea linkage segmented PUU copolymers. The obtained PUU copolymers in this work were named as PUU3-*n*, where *n* denotes the amount of IPDI used. (**B**) (**i**,**ii**) Illustrative depiction of the strong and stable H-bonding nanodomain cross-linked hydrogel network stemming from the aggregation of the hydrophobic multi-urea linkages. (**iii**) Photo image demonstrating ultra-strong and tough properties of the fully swollen hydrogel film with width of 10 mm and thickness of 1.0 mm. The film was dyed with Rhodamine B for easy observation. (**iv**) Scanning electron microscopy (SEM) image of the electrospinning PUU3-12 fibrous film from its DMF solution, 10 μm scale bar. (**v**) Microscopic image of the microfluidic-spinning PUU3-12-based hydrogel microfibers from its DMF solution, 100 μm scale bar. (**vi**,**vii**) The respective photo images of the original and stretched PUU3-12 hydrogel fiber. Adapted with permission from Reference [62]. Copyright 2017 Wiley-VCH.

There are several examples in literature showing the enhancement of the mechanical properties by H-bonding interactions. For example, Yang et al. used a linear polyurethane-urea (PUU) co-polymers with the aim of forming stable H-bonding interactions through modifying the polymer network with multiple urea linkages [62]. The synthesis was carried out using series of PUU co-polymers comprising mixtures of polyethylene glycol (PEG) and dimethylbutanoic acid (DMBA) as hydrophilic components, as well as several amounts of isophorone diisocyanate (IPDI) (Figure 9A). This preparation was prepared in a one-pot approach without any additional purification, which make it attractive for future mass production. 

Final formulations containing a constant mass ratio of 2:1 for PEG:DMBA but varying the IPDI amounts (8, 9, 12, 15 and 18) were also prepared. The presence of H-bonding interactions was studied by FITR spectroscopy and confirmed the formation of ordered and stronger electrostatic interactions due to the formation of H-bonded urea-urea linkages within the 3D polymer network (Figure 9B). The mechanical properties of PUU-based materials were characterized in terms of swelling (water contents varied from 55–75 wt.%) and rheology (the storage modulus *G’* remarkably increased as IPDI content (*n*) raised (35, 110, and 180 KPa for *n* = 8, 9, and 18, respectively). Additionally, hydrogels were proved to have high tensile strength (2–14 MPa), high toughness (10–60 MJ m^−3^), and elongation at break (600–1400%). Interestingly, these results were comparable to those found in living cartilage, ligaments and bond tissue and may serve as inspiring examples as biomaterials in other biomedical engineering applications. 

**Figure 10:**
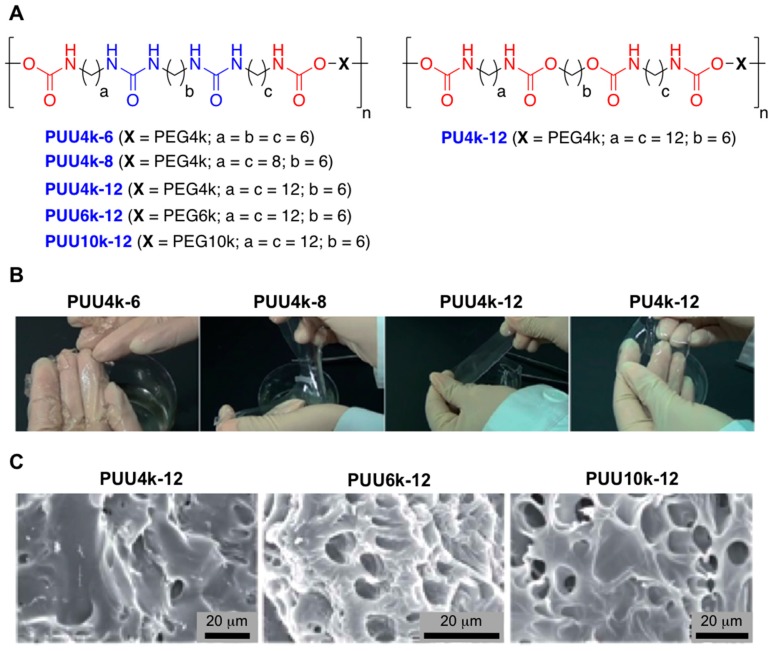
(**A**) Chemical structure of linear PUU and PU (PU4k-12) copolymers with various lengths of hydrophobic alkyl spacers and PEG chains. (**B**) Photo images of the PEG4k based PUU and PU supramolecular hydrogels. (**C**) SEM micrographs showing the cross-sections of the freeze-dried fully swollen PUUS hydrogels formed by PUU4k-12, PUU6k-12, and PUU10k-12 copolymers. Adapted with permission from Reference [63]. Copyright 2015 Royal Society of Chemistry.

Other representative examples combined H-bonding interactions along with hydrophobic forces. This strategy allowed Cui et al. to obtain stretchy and tough materials from a small library of chemically modified polyurethane-urea (PUU) co-polymers [63]. The resulting materials, which were obtained following one-pot polymerization strategies, were made of hydrophobic alkyl spacers series (C_6_, C_8_, and C_12_) and PEG chains of different lengths (4k, 6k, and 10k) (Figure 10A). The authors were able to measure the corresponding urea H-bonding formation of the modified PUU co-polymers from the C=O stretching peak values obtained by FT-IR spectroscopy when these polymer series were in contact with water. These measurements corroborated that co-polymers modified with short alkyl chains (C_6_ and C_8_ and PEG4k) displayed a mixture of free C=O (ca. 1720 cm^−1^) and a broad H-bonded C=O peaks (ca. 1670 cm^−1^). However, co-polymers made of long alkyl chains (C_12_) confirmed the presence of a broad intense peak due to the presence of urea-urea H-bonding interactions. Three PUU supramolecular hydrogels with various PEG residues of different lengths were prepared taking the effect of this long hydrophobic alkyl chain into account. The mechanical properties of the resulting materials were evaluated. These measurements confirmed fully swollen (*W*_H2O_ = 84–92 wt.%), strong (σ_b_ = 0.99–1.48 MPa) and tough (*W*_e_ = 4.2–7.55 MJ m^−3^) hydrogels with cyclic responsive shaped memory when subjected to certain stimuli (Figure 10B).

**Figure 11:**
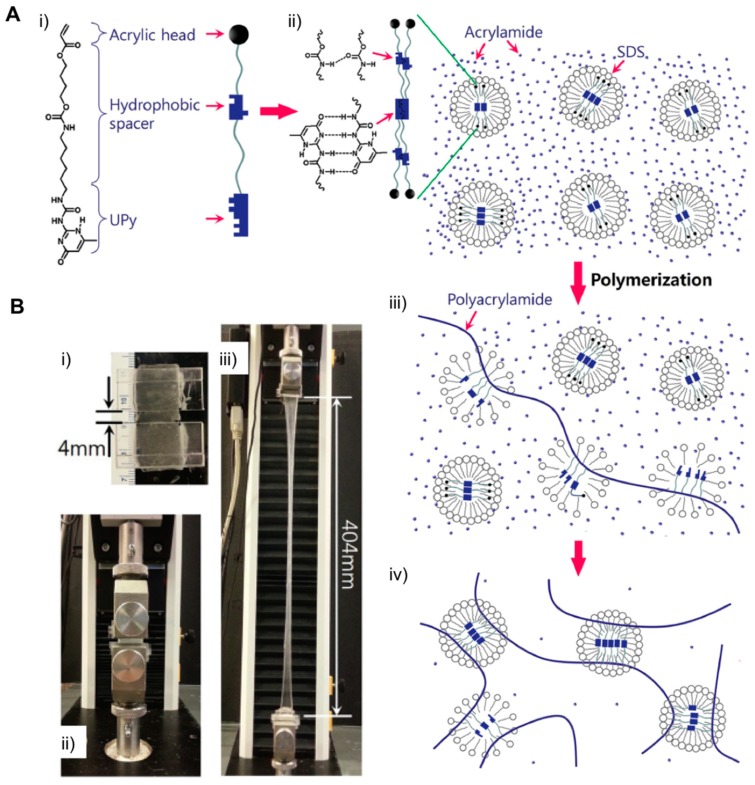
(**A**) Schematic of the micellar copolymerization of the UPyHCBA and acrylamide. (**i**) The structure of the UPyHCBA monomer; (**ii**) SDS micelles loaded with UPyHCBA in an aqueous acrylamide solution, and (**iii**,**iv**) micellar copolymerization of acrylamide and UPyHCBA loaded in SDS micelles. (**B**) (**i**) Hydrogel specimen for tensile testing. Upper and lower sections of the sample were sandwiched between two polystyrene plates using glue (Krazy Glue, KG585) to produce a 4 mm gauge length. The width and thickness of the specimen were 15.0 and 6.0 mm, respectively. A gel specimen prepared using 9.1 × 10^−3^ M UPyHCBA (**ii**) before and (**iii**) after stretching. Adapted with permission from Reference [64]. Copyright 2016 Wiley-VCH.

The use of synthetic cross-linkers was also reported with the aim of optimizing noncovalent entanglements within network structures. Jeon et al. described the synthesis of UPyHCBA, a novel cross-linker made of an acrylic head, a 2-ureido-4-pyrimidone and a hydrophobic alkyl spacer [64] (Figure 11A). This mixture in combination with SDS micelles afforded both hydrophobic interactions and the hydrogen bonds necessary to modulate, and therefore altered the properties of the 3D network in the presence of an aqueous acrylamide solution. The authors reported good mechanical properties in the material because of the interaction between UPyHCBA and SDS micelles. As a consequence, combining physical and dynamic hydrophobic cross-linking interactions promoted by SDS micelles in only one system resulted in a good strategy for getting stretchable and self-healable hydrogels. This was reflected in improving the elastic capacity of the hydrogel (up to 100 times if compared with its initial stage), as well as rapid self-healing ability, exhibiting full recovery of the hydrogel fracture (ca. 30 s) without any additional external energy (Figure 11B). Interestingly, the absence of SDS micelles reduced the anticipated H-bonding interactions and also contributed to decreasing the cross-link density due to the solvation effect exhibited by the hydrogel in aqueous solution.

**Figure 12:**
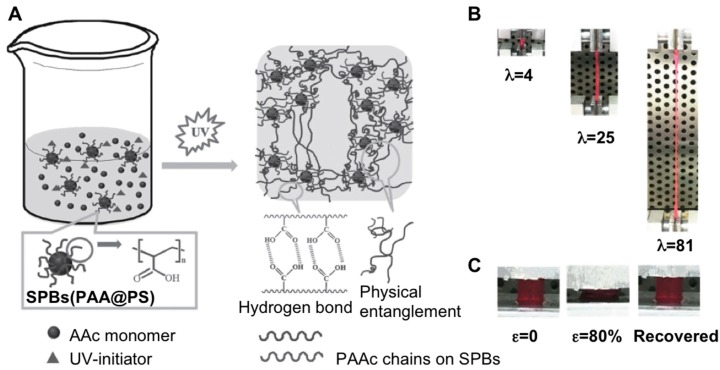
(**A**) Schematic illustration of the synthesis of PAAc/PAAc@PS hydrogels. (**B**) Pictures of S_0.1_A_0.4_ gels in tensile test. (**C**) Photographs of S_0.1_A_0.6_ gels in compressive test. Nomenclature of hydrogels: S*_n_*A*_m_*, where *n* and *m* are the weight contents of PAAc@PS solution (0.1 wt.%) and PAAc in the hydrogel, respectively. Adapted with permission from Reference [66]. Copyright 2017 Wiley-VCH.

Other kinds of H-bonding interactions were provided within a polymer network with the use of spherical polymer brushes (SPBs) [65] that are able to intermingle with PAAc chains in the same way as clay cross-linkers do. In this regard, Zhang et al. prepared physical cross-linked hydrogels based on PAAc/PAAc-grafted polystyrene (PAA@PS) by carrying out a photo-emulsion polymerization reaction and subsequent UV irradiation [66] (Figure 12A). Thus, as the PAAc content was increased, the authors were able to prepare a good number of hydrogels containing different porous structures which afforded different response to stimuli. For instance, these materials displayed great ability for elongation (up to 9.1 × 10^3^%) and toughness (up to 3.0 MJ m^−3^) in several tensile tests as the amount of PAAc was increased (Figure 12B,C). Further experiments also exhibited an equilibrium swelling ratio (up to 2.0 × 10^3^), fracture at break of up to 85%, as well as good self-recovery (compressive stress was up to 1.80 MPa). These positive responses were attributed to the presence of the H-bonding interactions based on PAAc-grafted polystyrene which was able to create a strong network when these materials were swelled in aqueous solvents. The mechanical properties were also studied by varying the weight content of PAAc@PS from 0.01 to 0.1 wt.%. This allowed the authors to increase both fracture stress (from 62.0 to 89 KPa) and compressive stress (from 1.0 to 1.6 MPa).

Host-guest complexation is another strategy for forming non-covalent interactions between polymer chains. The use of host-gest chemistries has attracted a lot of attention in biomedical research [67,68,69,70,71,72] and in particular, the use of cucurbiturils and cyclodextrins, which were proven to be effective macrocyclics for modulating the mechanical properties of supramolecular hydrogels. Some relevant examples were recently reported. Liu et al. carried out in situ photoinitiated polymerization reactions between polymerizable guest monomers like 1-benyl-3-vinylimidazolium and acrylamide along with cucurbit [8]uril (CB[8]), which acted as a macrocyclic host [73]. The resulting mixture was expected to lead to the formation of the supramolecular networks. This gelation process was studied in detail by increasing the monomer concentration (up to 2.0 M) but keeping the amount of the CB[8] (2.5 mol%) constant. Interestingly, high concentration of polymer chains increased the level of entanglements in the 3D network and stabilized the supramolecular network in the presence of noncovalent interactions. However, when gelation processes were carried out in the absence of the host molecule, viscous liquids were obtained. This strategy allowed the authors to carry out polymerization reactions with the aim of producing materials with extraordinary capacity of stretching (up to 100 times their initial length) and the ability to heal themselves at room temperature when CB[8] host molecules acted as dynamic cross-linkers within these 3D networks.

**Figure 13:**
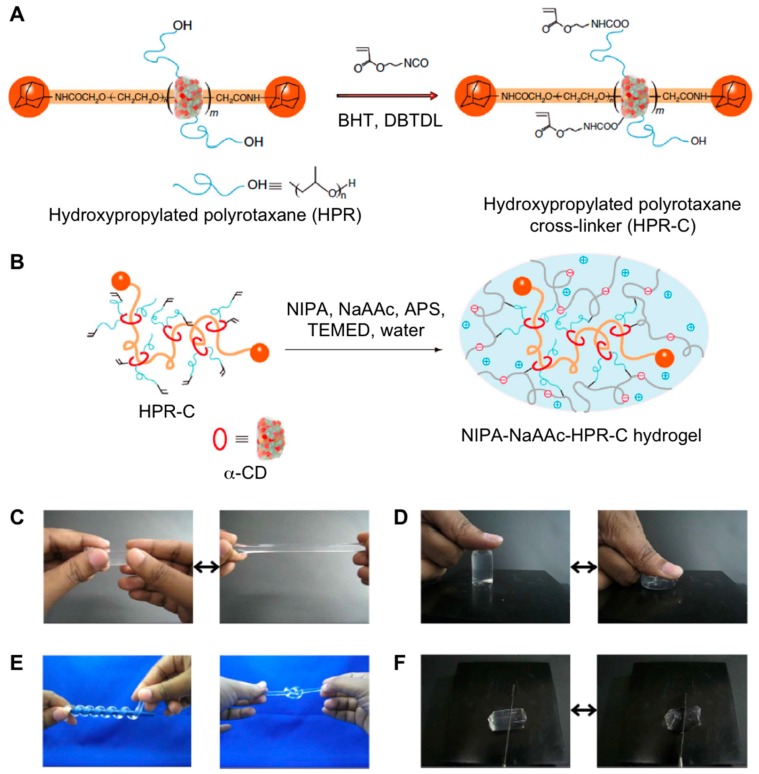
(**A**) Preparation of HPR-C from HPR, 2-acryloyloxyethyl isocyanate, dibutyltin dilaurate (DBTDL) (catalyst) and butyl hydroxyl toluene (BHT) (polymerization inhibitor) in DMSO. (**B**) Preparation of the NIPA-NaAAc-HPR-C hydrogel from HPR-C (cross-linker), NIPA (main monomer), NaAAc (comonomer), ammonium persulphate (APS) (initiator) and TEMED (accelerator) in water. (**C**) Elongated state of the NIPA-NaAAc-HPR-C hydrogel. (**D**) Compressed state of the NIPA-NaAAc-HPR-C hydrogel. (**E**) Coiled and knotted states of the NIPA-NaAAc-HPR-C hydrogel. (**F**) The NIPA-NaAAc-HPR-C hydrogel could not be easily cut with a knife. Adapted with permission from Reference [74]. Copyright 2014 Nature Publishing Group.

Other polyrotaxanes have been successfully used for preparing highly stretchable materials with good toughness. In this regard, Imran et al. were able to obtain polymeric networks made of sodium acrylic acid (NaAAc), *N*-isopropylacrylamide (NIPA) and α-cyclodextrin (α-CD), which was conjugated with PEG [74] (Figure 13A). The presence of this α-CD within the 3D network and their combination with the ionic monomer NaAAc, enabled the free movement of cyclodextrin through the polymeric chains. This gave the material the property of being highly stretchable with a range of 400 to 800% according to the original size, thereby reducing the brittleness of conventional hydrogels (Figure 13B). Interestingly, the authors found the stretching degree could be modulated depending on the cross-linker used like hydropropylated polyrotaxane (HPR) or MBAAm. The changes observed in the mechanical properties were susceptible to both temperature and pH giving rise to stimuli-sensitive materials that could be used for a good number of biomedical applications such as drug delivery systems, artificial muscles, and tissue engineering, among others.

## 3. Conclusions

Numerous strategies have been developed in order to improve the mechanical properties of hydrogels. In many cases, ordinary synthetic and natural materials are found to be too brittle due to the presence of moderate intermolecular forces among polymers chains. This causes the corresponding synthetic or natural materials to be more sensitive to external forces. This feature widely restricts their use in multiple applications. In this regard, the search for alternative approaches with the aim of improving stretchability, toughness, and self-healing abilities of synthetic hydrogels remain challenging issues. A good number of approaches have been taken in order to improve mechanical behaviors by designing mechanisms that can promote energy dissipation throughout hydrogel polymer networks after their exposure to external stimuli. Thus, the engineering of 3D networks based on dual network hydrogels, host-guest recognition, nanocomposite hydrogels, and. macromolecular microspheres, as well as the presence of both polymer chains’ hydrophobic association and H-bonding electrostatic interactions represent an opportunity to improve the mechanical strength of numerous hydrogels. These strategies helped create different possibilities of tailoring and launching synthetic hydrogels for multiple applications such as artificial muscle, cartilage, and tissue engineering, among other biomedical engineering processes. In this context, future works with these materials should address some important aspects, such as the biocompatibility (immune response) of these materials; degradation in vivo, and the fates of nanoparticles released from composite materials. Also, it would be useful to draw specific correlations, if any, between the polymer content or the features of a given particle-polymer system with the toughness in the resulting hydrogels. Finally, it should be emphasized that most of the highly tough materials described here have water contents below 90%. Thus, and especially for biomedical applications, besides achieving maximum toughness, extensibility, or compressive modulus, it is also desirable to develop strategies to reduce brittleness while maintaining a high degree of hydration.

## Abbreviations

AAc	acrylic acid
AAm	acrylamide
AIBN	2,2′-azobis(2-methylpropionitrile)
BA	butyl acrylate
BHT	butyl hydroxyl toluene
CaGP	2-glycerol phosphate
CB[8]	cucurbit[8]uril
α-CD	α-cyclodextrin
3D	tridimensional
DAC	2-(dimethylamino)ethylacrylatemethyl chloride
DC	double cross-linked (DBTDL: dibutyltin dilaurate
DCPA	dicyclopentyl acrylate
D-Gel	dual-cross-linked hydrogels
DLS	dynamic light scattering
DMBA	dimethylbutanoic acid
DN	double-network
DPC	dual physically cross-linked
EDPOA	ethyl-2-[4-(dihydroxyphosphoryl)-2-oxabutyl]-acrylate
G-6-P	glucose-6-phosphate
HMA	hexadecyl methacrylate
HPR	hydropropylated polyrotaxane
IPDI	isophorone diisocyanate
IPN	interpenetrating polymer network
KPS	potassium peroxodisulfate
MBAAm	*N*,*N*′-methylene-bis(acrylamide)
M-Gel	mono-cross-linked hydrogels
MMSPH	macromolecular microspheres
MPS	3-methacryloxypropyltrimethoxysilane
NaAAc	sodium acrylic acid
NaMMT	sodium montmorillonite
NCHs	nanocomposite hydrogels
NIPA	*N*-isopropylacrylamide
LMWG	low molecular weight gelators
PAAc	polyacrylic acid
P(AAm-*co*-AAc)	poly(acrylamide-*co*-acrylic acid)
P(AAm-*co*-LMA)	poly(acrylamide-*co*-laurylmethacrylate)
PAAm-*l*-MBAm	Polyacrylamide-*l*-*N*,*N*′-methylenebis-(acrylamide)
PDMA-*l*-TEG	poly-*N*,*N*-dimethyl acrylamide-*l*-TEG
PEG	polyethylene glycol
PF127	pluronic F127
PHEA-*l*-TEG	triethylene glycol dimethacrylate
PNIPAm	poly(*N*-isopropyl acrylamide)
PUU	polyurethane-urea
Q-CNCs	quaternized cellulose nanocrystals
TEMED	*N*,*N*,*N*,*N*-tetramethylethylenediamine
SEM	scanning electron microscopy
SDS	sodium dodecyl sulfate
SMA	stearyl methacrylate
SPBs	spherical polymer brushes
TEM	transmission electron microscopy
